# Supplementary figures and images for: Differential restriction of chikungunya virus in primary human cardiac endothelial cells occurs at multiple steps in the viral life cycle
Source: PLoS Negl Trop Dis. 2025 Mar 10;19(3):e0012534. doi: 10.1371/journal.pntd.0012534 (PMC11918386; doi:10.1371/journal.pntd.0012534)

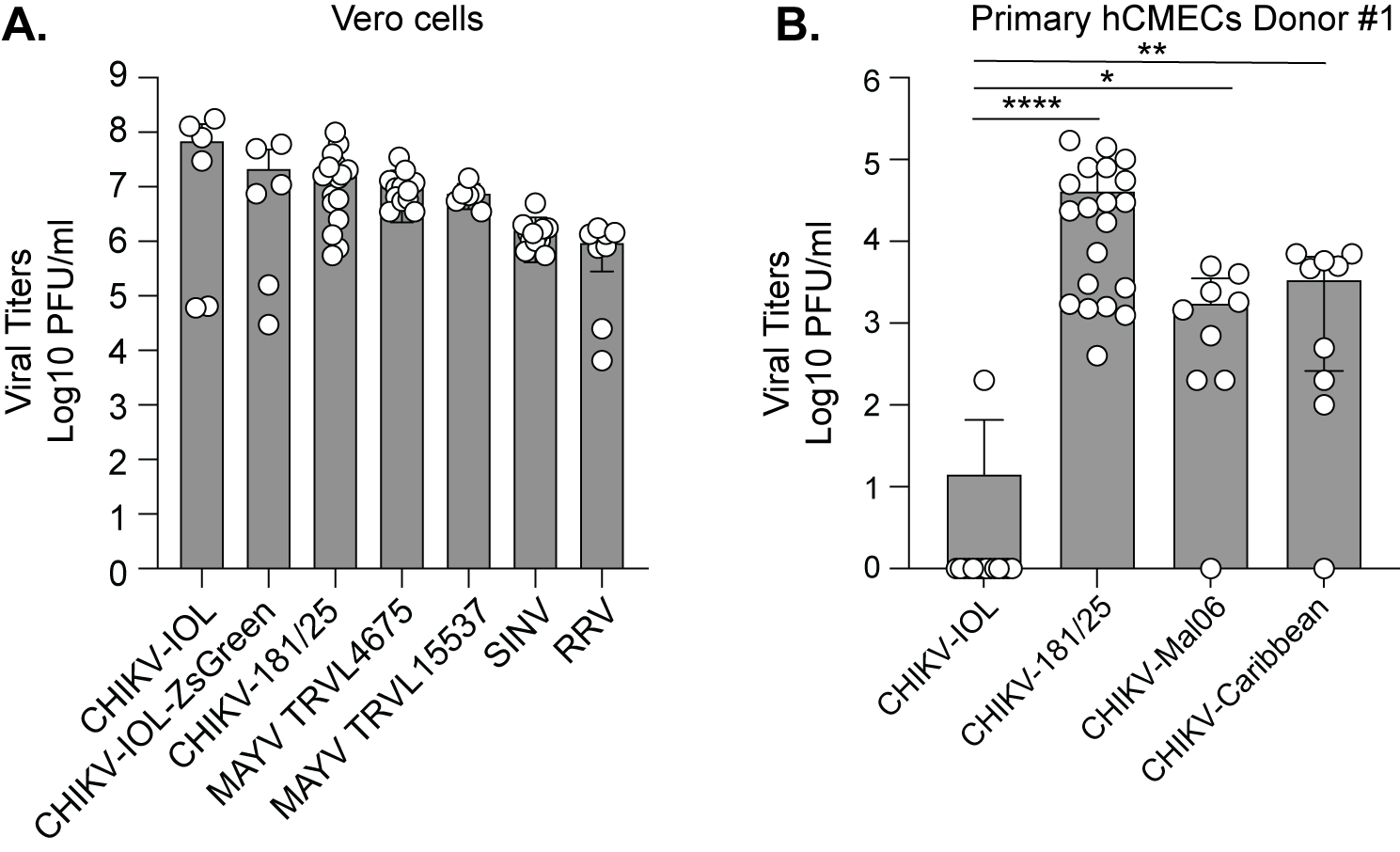

Supplement: S1 Fig — (A) Vero cells or (B) hCMECs were infected with each virus at an MOI of 0.1. 48 hours post infection the supernatant was collected, and infectious particles were quantified by plaque assay. For Vero cells, 3-8 independent experiments (n = 6-16). For hCMECs, three independent experiments (n = 9). Data represent the mean and SD. Kruskal-Wallis test. * p < 0.05, ** p < 0.01, **** p < 0.0001. (TIF) [file pntd.0012534.s001.tif]

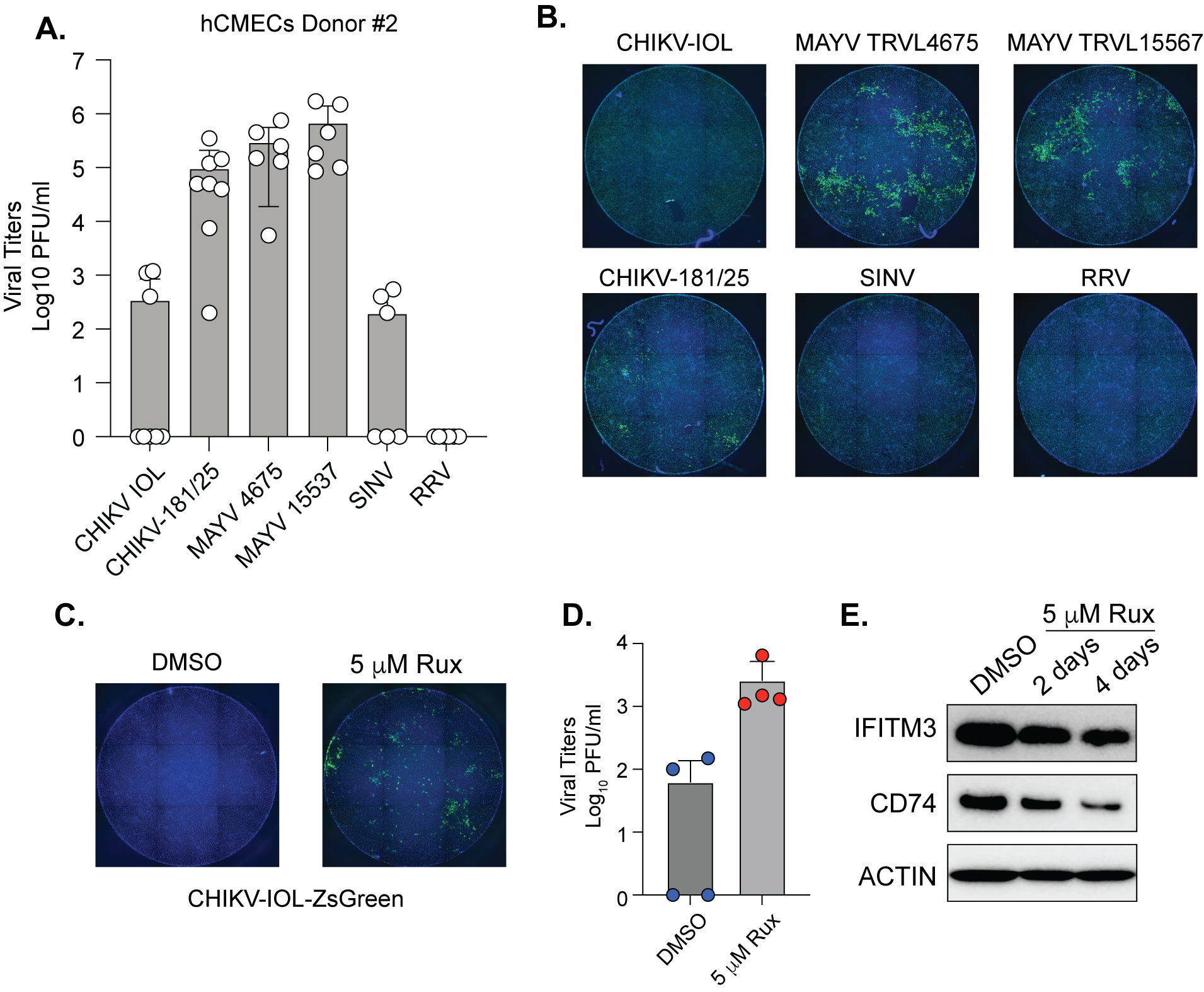

Supplement: S2 Fig — (A) hCMECs were infected with each virus at a MOI of 0.1. 48 hours post infection, infectious virus in the supernatant was quantified by plaque assay. Three independent experiments (n=6). Data represent the mean and SD. (B) At 48 hours cells were fixed and stained with DAPI and polyclonal antibody for capsid. Representative images of two independent experiments shown. (C) hCMECs were treated with DMSO or ruxolitinib (Rux) for 48 hours, infected with CHIKV-IOL-ZsGreen an at MOI of 0.1, and cells fixed 48 hours post infection. (D) Viral titers in the supernatant from cells in (C) quantified by plaque assay. Two independent experiments (n=4). (E) hCMECs were treated with DMSO or Rux for the indicated time, collected in lysis buffer, and proteins analyzed by SDS-PAGE and immunoblotting. Blots represent one of two independent experiments. (TIF) [file pntd.0012534.s002.tif]
